# Occurrence and Type of Adverse Events During the Use of Stationary Gait Robots—A Systematic Literature Review

**DOI:** 10.3389/frobt.2020.557606

**Published:** 2020-11-16

**Authors:** Jule Bessler, Gerdienke B. Prange-Lasonder, Robert V. Schulte, Leendert Schaake, Erik C. Prinsen, Jaap H. Buurke

**Affiliations:** ^1^Roessingh Research and Development, Enschede, Netherlands; ^2^Department of Biomedical Signals and Systems, University of Twente, Enschede, Netherlands; ^3^Department of Biomechanical Engineering, University of Twente, Enschede, Netherlands

**Keywords:** robot-assisted gait training, adverse event (AE), safety, physical human-robot interaction (pHRI), injuries (MeSH), stationary gait robots, rehabilitation robotics

## Abstract

Robot-assisted gait training (RAGT) devices are used in rehabilitation to improve patients' walking function. While there are some reports on the adverse events (AEs) and associated risks in overground exoskeletons, the risks of stationary gait trainers cannot be accurately assessed. We therefore aimed to collect information on AEs occurring during the use of stationary gait robots and identify associated risks, as well as gaps and needs, for safe use of these devices. We searched both bibliographic and full-text literature databases for peer-reviewed articles describing the outcomes of stationary RAGT and specifically mentioning AEs. We then compiled information on the occurrence and types of AEs and on the quality of AE reporting. Based on this, we analyzed the risks of RAGT in stationary gait robots. We included 50 studies involving 985 subjects and found reports of AEs in 18 of those studies. Many of the AE reports were incomplete or did not include sufficient detail on different aspects, such as severity or patient characteristics, which hinders the precise counts of AE-related information. Over 169 device-related AEs experienced by between 79 and 124 patients were reported. Soft tissue-related AEs occurred most frequently and were mostly reported in end-effector-type devices. Musculoskeletal AEs had the second highest prevalence and occurred mainly in exoskeleton-type devices. We further identified physiological AEs including blood pressure changes that occurred in both exoskeleton-type and end-effector-type devices. Training in stationary gait robots can cause injuries or discomfort to the skin, underlying tissue, and musculoskeletal system, as well as unwanted blood pressure changes. The underlying risks for the most prevalent injury types include excessive pressure and shear at the interface between robot and human (cuffs/harness), as well as increased moments and forces applied to the musculoskeletal system likely caused by misalignments (between joint axes of robot and human). There is a need for more structured and complete recording and dissemination of AEs related to robotic gait training to increase knowledge on risks. With this information, appropriate mitigation strategies can and should be developed and implemented in RAGT devices to increase their safety.

## Introduction

Robot-assisted gait training (RAGT) is frequently used in rehabilitation to promote walking function in individuals with various disabilities, such as stroke, spinal cord injury (SCI), or cerebral palsy. The rates of disability, e.g., as a result of chronic stroke, are rising due to population aging. According to the World Health Organization, the proportion of the world's population aged over 60 years will increase drastically from 12% in 2015 to 22% in 2050 (World Health Organization, 2018). This leads to an increasing amount of persons with chronic walking disabilities that will in turn lead to a lack of skilled physical therapists.

Robotic gait trainers can be used for various patient groups to provide them with high-intensity gait training. While traditional gait training on a treadmill is associated with high physical strain on the therapists and a need for two to three therapists per patient, robotic gait trainers have the advantage of reducing the time and effort required from the therapist. As a result, they potentially allow for longer or more frequent sessions of high-intensity gait training for the patient (Hesse et al., 2003).

There are different types of robotic gait trainers. Overground gait trainers include ambulatory exoskeletons, such as the ReWalk (Argo Medical Technologies Ltd., Israel), Ekso GT (Ekso Bionics, USA), HAL (Cyberdyne, Japan), REX (Rex Bionics, New Zealand), and Indego (Parker Hannifin Corp., USA). Stationary gait trainers can be divided into two subcategories: exoskeleton-type devices and end-effector-type devices. Exoskeleton-type devices usually consist of a treadmill, an overhead harness for body-weight support (BWS), and a lower limb exoskeleton fixed to a frame. Examples of exoskeleton-type devices are the Lokomat (Hocoma, Switzerland), AutoAmbulator (Motorika, USA), RoboGait (Bama Technology, Turkey), Walkbot (P&S Mechanics, South Korea), and NX-A3 (Guangzhou YiKing Medical Equipment Industrial, China). End-effector-type devices, such as the G-EO system (Reha-Technology, Switzerland), LokoHelp (Woodway, Germany), Gait Trainer GT II (Reha Stim Medtec, Germany), and THERA-Trainer Lyra (medica Medizintechnik, Germany), consist of an overhead BWS and robotic end-effectors that are attached to the patient's feet and are moved along reference trajectories of normal walking.

The advantages of RAGT with regard to time and physical effort required by the therapist are obvious (Mehrholz et al., 2017). However, the mechanical power of the robots in combination with the close physical connection with the patient inevitably introduces safety issues. The robot is attached to the patient's limbs, which can lead to dangerous interaction forces. Safe ranges of normal and shear forces that can be applied to a patient during training with a robot are yet to be defined. While recent research has focused on safe limit values in collision situations of physical human–robot interaction (pHRI) (Haddadin et al., 2007; Behrens and Elkmann, 2014), situations of continuous contact are challenging to assess. This is mostly due to a lack of reliable measurement methods, especially concerning shear forces. Much effort has recently been put into the development of those measurement methods (Lenzi et al., 2011; Sugiura et al., 2012; Makino et al., 2013; Castellini and Ravindra, 2014; Ito et al., 2014; Tamez-Duque et al., 2015; Wilkening et al., 2016; Alavi et al., 2017; Sadarangani et al., 2017). A method that can be considered as the gold standard for measuring normal and tangential forces is the load cell. However, these sensors are rather bulky and expensive, which are possible reasons why many studies implement force sensitive resistors to assess the interaction between a human and a robotic, orthotic or load-carrying device (Castellini and Ravindra, 2014; Tamez-Duque et al., 2015; Sadarangani et al., 2017). Drawbacks of force sensitive resistors include a typically non-linear transfer function, as well as sensitivity to changes of humidity and surface curvature (Castellini and Ravindra, 2014; Wettenschwiler et al., 2015), which are highly relevant during the measurement of prolonged human–robot interaction (HRI) between skin and cuff. A number of studies have focused on developing and implementing alternative sensing devices, such as optical sensors (Lenzi et al., 2011; Sugiura et al., 2012; Makino et al., 2013), vision-based tactile sensors (Ito et al., 2014), and pneumatic padding (Wilkening et al., 2016; Alavi et al., 2017); however, these methods are still in the research state.

Besides the much needed safe limit values for continuous HRI, simplifying the safety evaluation process is another contributor to improving safety in collaborative and rehabilitation robotics. This is, for example, done in the COVR project (www.safearoundrobots.com) by providing various structured tools for robot developers, including establishing the best practices for safety-related measurements and promoting the development and application of unified safety testing procedures. As a first step in this, specifically regarding rehabilitation robots, risks, and needs covering all aspects of continuous patient–robot interaction should be assessed in a structured way, which in turn should inform the development of relevant measurement methods. Therefore, adverse events (AEs) of existing rehabilitation robots need to be taken into account and associated risks need to be identified. A recent review assessed the aspects of risk management and the occurrence of AEs in overground exoskeletons (He et al., 2017). Both the FDA (Food and Drug Administration of the United States) database MAUDE (Manufacturer and User Facility Device Experience) and peer-reviewed publications including any of the overground exoskeleton device names mentioned above were searched for AEs during the usage of exoskeletons. The review found, among other AEs, a number of device malfunctions, skin and tissue damages, and two incidences of bone fractures. Both incidences of bone fractures were attributed to misalignment of the device causing a discrepancy between human joint axis and robot joint axis. This is an indication for the need for extensive post-market surveillance and appropriate testing methods for safety of robotic gait rehabilitation devices.

A recent Cochrane review assessing the clinical effects of electromechanical-assisted training for walking after stroke (Mehrholz et al., 2017) also collected information on any AEs reported in those studies. The most frequently documented adverse effects and reasons for dropout were pain and skin breakdown. In light of obvious differences between stationary RAGT and overground exoskeletons, such as the use of BWS in stationary RAGT compared with crutches in overground exoskeletons to decrease the risk of falls, and professional supervision compared with oversight by a trained family member, it seems straightforward to assume that AEs in stationary RAGT are less frequent and less severe than those in overground exoskeletons due to its controlled environment. However, there is insufficient structured information available on the occurrence of AEs in stationary RAGT. Moreover, there currently is no European equivalent to the US MAUDE database in operation, and other parts of the world have in turn different processes (Mishra, 2017), which makes it difficult to find reliable worldwide information on the frequency and severity of AEs.

Therefore, this paper presents a systematic literature review of AEs that occurred during training with stationary robotic gait trainers. We hypothesized that there are incidences of skin breakdown and bone fractures in RAGT and further expected that the reporting of these events is lacking detail. We searched both bibliographic and full-text literature databases for peer-reviewed articles describing the outcomes of RAGT and specifically mentioning AEs. From this, we extracted information about AEs and their reporting, with the objective to get an overview of the occurrence and type of AEs in stationary robotic gait trainers and identify particular risks involved.

## Methods

### Search Strategy and Data Sources

We conducted an electronic database search in relevant bibliographic (PubMed, Scopus, Web of Science) and full-text databases (IEEE Xplore Digital Library, SpringerLink, ScienceDirect, SAGE Publications, AHA Journals) from inception to mid-June 2019. We used the following search terms for all databases:

- Electro-mechanical, electromechanical, robotics, robot-assisted, robotics-assisted
- Exercise therapy, rehabilitation, training
- Gait, walk, walking, step, stepping, locomotor, locomotion
- Bodyweight-supported treadmill training, locomotor training, Lokomat, Gangtrainer (GT), G-EO, WALKBOT, LokoHelp
- Adverse, skin breakdown, skin lesion, skin sore, pressure sore, discomfort, abrasion.

The complete search strategy used in PubMed can be found in the Appendix (Supplementary Material). This search was adapted to suit the other databases, and we searched full text (where available), title, abstract, and keywords. Reference lists of included articles were scanned for potentially relevant additions.

### Study Selection

The criteria that were applied for study selection can be found in Table 1. We did not apply criteria in terms of study design, population, or comparators as we aimed to find all available information on AEs in stationary robotic gait training with humans. After exclusion of duplicate entries, the titles were screened by two reviewers independently (GP and JB). Following that, the abstracts of the remaining studies were screened by EP and JB, and in the third step, the full texts were screened by RS and JB. A third reviewer could be consulted in case of a disagreement between the two respective reviewers (EP for title screening and GP for abstract and full-text screening). Title and abstract screening were performed using a web-based tool (Ouzzani et al., 2016).

**Table 1:** Inclusion and exclusion criteria.

**Criteria**	**Inclusion criteria**	**Exclusion criteria**
1	Articles must be peer-reviewed (full) papers	Conference abstracts and other non-peer-reviewed articles were excluded
2	Articles must be trials with human subjects	All articles that were not trials including human subjects (e.g., literature reviews, study protocols, animal studies) were excluded
3	Articles must address robotic-assisted stationary gait training Either exoskeleton-type or end-effector-type Person standing upright, doing stepping movements Being attached to the lower extremity Stationary	Articles addressing other technologies (e.g., surgical robots, overground exoskeletons, upper-limb robots) were excluded
4	Articles must include a specific statement about AE (this can also be a statement saying that no AE occurred)	Articles not including any statement about AE related to the robotic gait training were excluded
5	Articles must be available in the English language	Articles written in other languages were excluded

### Data Extraction and Analysis

As this systematic review's main aim was to collect and analyze AEs in RAGT, we did not perform a methodological quality judgment. We employed the PRISMA reporting guidelines (Moher et al., 2009) as far as they were applicable to this review. Restrictions of their applicability were due to the fact that this review does not focus on the clinical effects of an intervention. For collecting relevant data from all included studies, we developed a structured table. The data categories that the studies were screened for are:

1. Subject characteristics
2. Training device
3. Study design
4. Description of AEs and dropouts.

We collected information on the number of subjects performing gait training, age, diagnoses, time since onset, and severity. Since the diagnoses varied strongly, no overarching measure for disease severity or disease stage could be defined to describe the functional level or chronicity. The study design type as well as the number and duration of sessions were noted. Device types (exoskeleton-type, end-effector-type, soft exosuit) and names were collected. Moreover, we screened for information on the amount of BWS and the type of HRI. Types of HRI, such as active, passive, and assistive, were based on the review by Basteris et al. (2014).

Regarding AEs, we collected the number of studies reporting the presence of AEs, number of affected study participants, methods used to detect AEs, as well as numbers and types of AEs. Where it became apparent that several studies reported on the same trial (same intervention and same patients), we excluded any double reports to avoid bias. The description of AEs was assessed for completeness. An AE description was considered as complete whenever it included (1) a description of the AE itself including the number of occurrence, (2) the number of subjects affected, and (3) the intervention during which the AE occurred. A statement that no AE occurred was rated as incomplete if it contained contradictory information or was lacking information [e.g., only part of the intervention considered, only referring to serious adverse events (SAEs)]. We only collected AEs that were related to RAGT. When an event was described by the authors as unrelated to the intervention, it was not included in the data for this review. We did, however, include events with unspecified causes.

For better comparison between studies, AE type and severity were categorized as follows. For the type of AEs, we used the categories soft tissue-related (e.g., skin reddening, lesions, bruises, discomfort from harness), musculoskeletal (e.g., joint pain, muscle pain, bone fractures), and physiological (e.g., blood pressure changes). Events that matched neither of these categories (e.g., headache, fear) were classified as other. Severity of AEs was classified as mild, moderate, or severe (adapted from Borggraefe et al., 2010; U.S. Department of Health Human Services, 2017):

- Mild: event is noticeable but easily tolerable. No medical intervention is needed, and treatment does not have to be interrupted or only for a short rest (e.g., minor discomfort, reddening)
- Moderate: event interferes with activities or treatment but can be managed by simple measures. No long-term effects (e.g., skin lesions without complications)
- Severe: event is incapacitating and requires medical attention/treatment, and normal treatment cannot be continued (e.g., bone fractures, skin lesions with complications)

If there was no description of an interruption of training or any other indication of a more severe event, the AE was assumed to be mild. Where there was no description of the AE that allowed us to conclude the severity, it was counted as unknown. Note that a severe AE as classified in this study does not automatically constitute an SAE according to the definition of the Medical Device Regulation (Regulation (EU) 2017/745 of the European Parliament of the Council of 5 April 2017 on medical devices, amending Directive 2001/83/EC, Regulation (EC) No 178/2002 and Regulation (EC) No 1223/2009 and repealing Council Directives 90/385/EEC and 93/42/EE, 2017). However, any SAE would be counted as severe in this review. To compare the severity of different AE types and between different devices or device types, we rated mild AEs with a severity of 1, moderate AEs with a severity of 4, and severe AEs with a severity of 10 and calculated the overall severity per device and per AE type as follows:

$$\begin{array}{lll} severity_{overall} & = & \frac{1\cdot n_{mild}+4\cdot n_{moderate}+10\cdot n_{severe}}{n_{total}-n_{unknown}}, \end{array}$$

where *n*_*mild*_ is the number of mild AEs, *n*_*moderate*_ is the number of moderate AEs, *n*_*severe*_ is the number of severe AEs, *n*_*total*_ is the total number of reported AEs, and *n*_*unknown*_ is the number of AEs with unknown severity level.

The classes of AE severity and their ratings (1 for mild, 4 for moderate, and 10 for severe) are chosen arbitrarily based on the authors' experience and judgment. They are not validated and are used solely to get a rough estimate of severities for comparisons between device types or AE types.

We performed Pearson's chi-squared tests of independence (MATLAB®, version 2019b, MathWorks, Natick, Massachusetts, USA) to investigate whether (1) devices (e.g., Lokomat, GT) are associated with AE types (e.g., soft tissue-related AEs, musculoskeletal AE), (2) device types (e.g., exoskeleton-type, end-effector-type) are associated with AE types, (3) AE types are associated with severity level (i.e., mild, moderate, severe), (4) devices are associated with severity level, and (5) device types are associated with severity level. We employed a significance level of 5%.

## Results

### Study Selection

**Figure 1:**
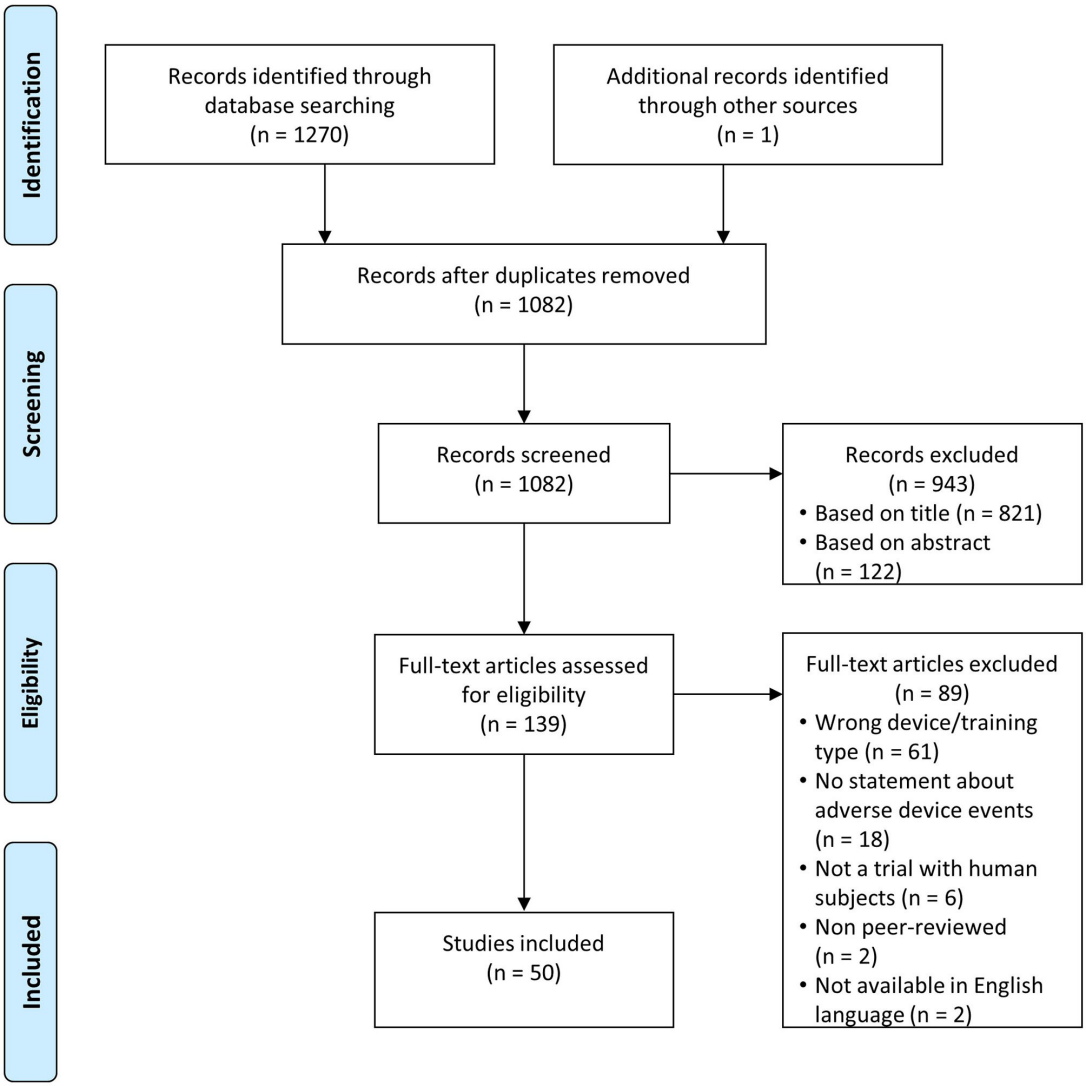
Flow diagram study identification based on Moher et al. (2009).

We identified 1,081 unique records through database searching and one addition through reference searching (Figure 1). Of those, 139 records remained after title and abstract screening, of which 50 met the inclusion criteria and were analyzed (Husemann et al., 2007; Mayr et al., 2007; Freivogel et al., 2008, 2009; Lo and Triche, 2008; Ng et al., 2008; Hesse and Werner, 2009; Borggraefe et al., 2010; Chin et al., 2010; Lo et al., 2010; Geroin et al., 2011; Morone et al., 2011; Turiel et al., 2011; Benito-Penalva et al., 2012; Carda et al., 2012; Gizzi et al., 2012; Picelli et al., 2012, 2015; Vaney et al., 2012; Geigle et al., 2013; Kelley et al., 2013a,b; Aach et al., 2014; Labruyère and van Hedel, 2014; Nilsson et al., 2014; Stoller et al., 2014, 2015; Asbeck et al., 2015; Filippo et al., 2015; Ochi et al., 2015; Schoenrath et al., 2015a,b; Sczesny-Kaiser et al., 2015, 2017; Wu et al., 2015; Chua et al., 2016; Forrester et al., 2016; Ikumi et al., 2016; Kumru et al., 2016a,b; Aurich-Schuler et al., 2017; Bae et al., 2017; Chisholm et al., 2017; Esquenazi et al., 2017; Grasmücke et al., 2017; Jansen et al., 2017, 2018; Kim et al., 2019; Straudi et al., 2019; Tanaka et al., 2019). We identified some studies with overlap in patients (Kelley et al., 2013a,b; Aach et al., 2014; Stoller et al., 2014, 2015; Sczesny-Kaiser et al., 2015; Jansen et al., 2017, 2018) and excluded double reports in the analysis of subject numbers and AE numbers.

### Patients and Devices

The included studies described RAGT in 985 subjects of which 14 were healthy individuals, 341 SCI patients, 326 stroke patients, 42 traumatic brain injury patients, 67 cerebral palsy patients, 74 Parkinson's disease patients, 76 multiple sclerosis patients, 15 cardiac patients, and 30 patients with other diagnoses. Two of the included studies focused on children and adolescents (Borggraefe et al., 2010; Aurich-Schuler et al., 2017). The identified studies reported on gait training in 10 different devices: Lokomat (489 subjects in 27 studies), Gait Trainer GT (301 subjects in 8 studies; 244 subjects trained in GT II, 24 in GT I, and for 33 subjects the model was not specified), HAL (108 subjects in 9 studies), MorningWalk (25 subjects in 1 study), LokoHelp (22 subjects in 2 studies), Anklebot (14 subjects in 1 study), Gait-Assistance Robot GAR (13 subjects in 1 study), G-EO (7 subjects in 1 study), a soft exosuit (5 subjects in 1 study), and PH-EXOS (1 subject in 1 study). One study reported the use of both Lokomat and G-EO, and one study reported the use of both Lokomat and GT.

### Adverse Events

Of the 50 included studies, 18 reported AEs, and 32 reported that there were no AEs (Table 2). The information on AEs was rated as incomplete in 8 (16%) of the 50 studies. In the studies with reported AEs, 78% of the AE descriptions were complete, whereas in the studies without reported AEs, 88% of the descriptions were complete. The dropout rate was 7% of the participants in both groups. Studies with AEs had 16 participants on average, and studies without AEs had 22 participants on average. Apart from the fact that none of the studies with healthy participants reported AEs, there were no striking differences in subject characteristics. Both age and diagnoses were comparable. Studies with AEs were less frequently randomized controlled trials (39 compared with 50% of studies without AEs) and were more likely to be case reports or case series, some of which were focused on reporting AEs (Geigle et al., 2013; Kelley et al., 2013b; Filippo et al., 2015). Concerning devices involved, 44% of the Lokomat studies, 33% of the HAL studies, and 13% of the GT studies reported AEs. There were no AEs reported for MorningWalk, Anklebot, Gait-Assistance Robot GAR, the soft exosuit, or PH-EXOS. The range of BWS in studies reporting AEs was between 0 and 100% of body weight, whereas it was between 0 and 50% of body weight in the studies reporting no AEs.

**Table 2:** Overview of studies with adverse events vs. studies without adverse events.

	**Studies that reported adverse events**	**Studies that reported no adverse events**
Number of studies	18	32
Completeness of AE description (complete/incomplete)	14/4	28/4
Number of subjects performing RAGT	291	694
Dropouts	19 dropouts in 6 studies, 0 lost to follow-up, 1 not stated	47 dropouts in 8 studies, 6 lost to follow-up in 4 studies, 3 not stated
Diagnosis	SCI, TBI, CP, stroke, PD, MS, other	SCI, TBI, CP, stroke, PD, MS, cardiac, other, healthy
Age (mean [SD of means])	42 (19)	53 (12)
Months since onset (range)	[0;276]	[1;420]
Level of severity	Mild to severe (FAC 0–4, ASIA A–D)	Mild to severe (FAC 0–5, ASIA A–D)
Study design type	7 RCT (2 pilot), 3 longitudinal uncontrolled, 2 longitudinal repeated measure (1 randomized, 1 controlled), 1 retrospective review of data, 4 case reports, 1 case series	16 RCT (2 pilot, 3 repeated measures), 9 longitudinal uncontrolled (2 pilot), 4 cross-sectional repeated measure (1 pilot), 2 longitudinal controlled, 1 longitudinal repeated measure
Number of studies per device	12 Lokomat^a^, 3 HAL^b^, 2 LokoHelp^c^, 1 GT^d^, 1 G-EO^e^	15 Lokomat^f^, 6 HAL^g^, 7 GT^h^, 1 soft exosuit^i^, 1 Anklebot^j^, 1 Morning Walk^k^, 1 GAR^l^, 1 PH-EXOS^m^
Number of sessions per participant (range)	[4;60]	[1;179]
Training duration per session (range) [min]	[6;60]	[0.5;45]
Total duration of training period (range) (days)	[8;84]	[1;365]
BWS (range)	(0% body weight; 100% body weight)	[0% body weight; 50% body weight]
Device types	15 exoskeleton, 4 end-effector	24 exoskeleton, 8 end-effector, 1 exosuit
Types of HRI	Assistive, active, passive	Assistive, active, passive, path guidance, resistive

*SCI, spinal cord injury; TBI, traumatic brain injury; CP, cerebral palsy; PD, Parkinson's disease; MS, multiple sclerosis; SD, standard deviation; FAC, functional ambulation category; ASIA, American Spinal Injury Association; RCT, randomized controlled trial*.

a *Husemann et al. (2007), Borggraefe et al. (2010), Chin et al. (2010), Carda et al. (2012), Vaney et al. (2012), Geigle et al. (2013), Kelley et al. (2013a,b), Stoller et al. (2014, 2015), Filippo et al. (2015), and Esquenazi et al. (2017)*.

b *Nilsson et al. (2014), Ikumi et al. (2016), and Jansen et al. (2018)*.

c *Freivogel et al. (2008, 2009)*.

d *Morone et al. (2011)*.

e *Esquenazi et al. (2017)*.

f *Mayr et al. (2007), Lo and Triche (2008), Lo et al. (2010), Turiel et al. (2011), Benito-Penalva et al. (2012), Gizzi et al. (2012), Labruyère and van Hedel (2014), Schoenrath et al. (2015a,b), Kumru et al. (2016a,b), Aurich-Schuler et al. (2017), Bae et al. (2017), Chisholm et al. (2017), and Straudi et al. (2019)*.

g *Aach et al. (2014), Sczesny-Kaiser et al. (2015, 2017), Grasmücke et al. (2017), Jansen et al. (2017), and Tanaka et al. (2019)*.

h *Ng et al. (2008), Hesse and Werner (2009), Geroin et al. (2011), Benito-Penalva et al. (2012), Picelli et al. (2012, 2015), and Chua et al. (2016)*.

i *Asbeck et al. (2015)*.

j *Forrester et al. (2016)*.

k *Kim et al. (2019)*.

l *Ochi et al. (2015)*.

m *Wu et al. (2015)*.

**Figure 2:**
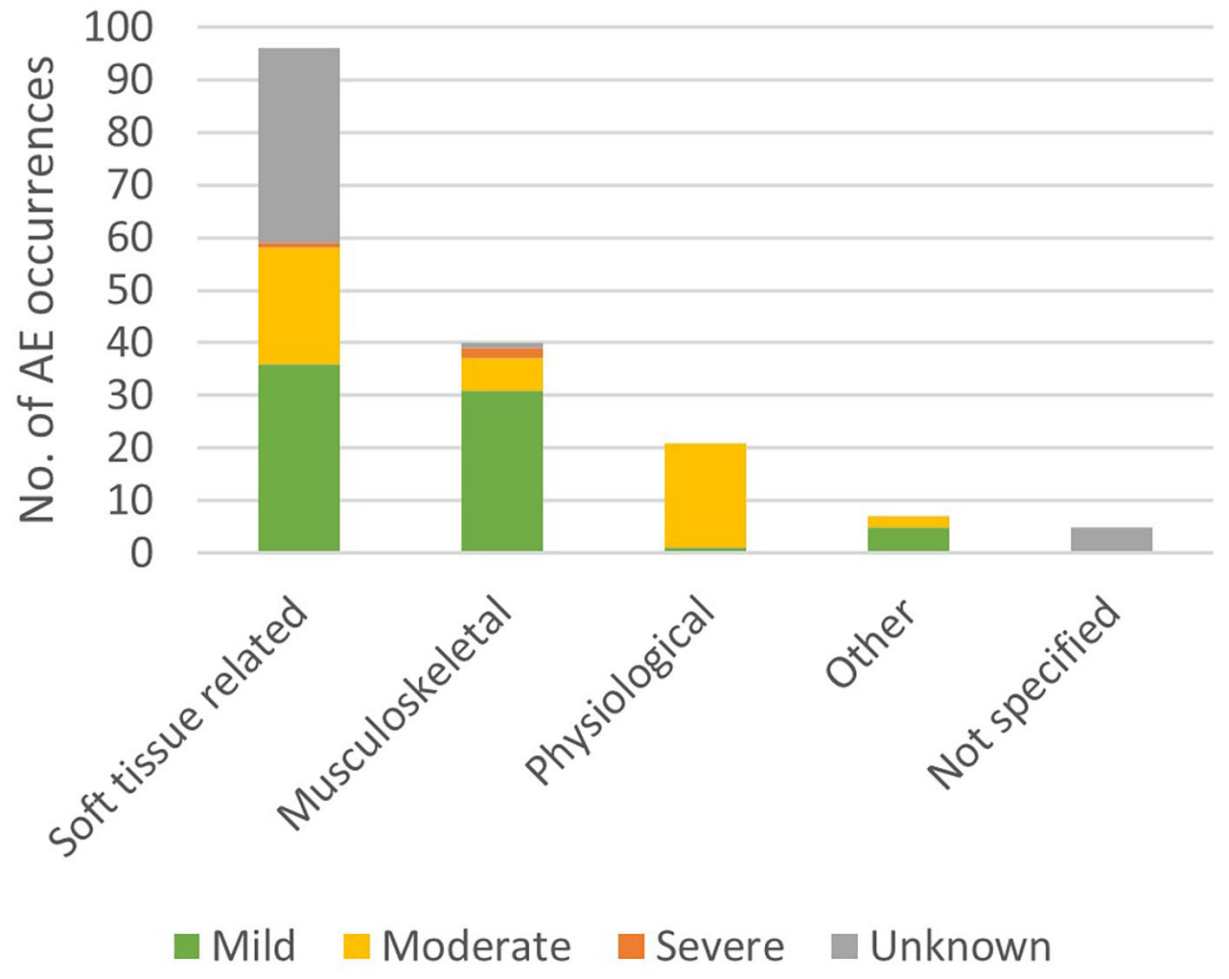
Occurrences of adverse event severities per adverse event types.

The AE descriptions from the 18 studies that did report AEs are collected in Table 3. The most frequently reported AEs were changes to the skin or soft tissue (more than 47 occurrences in 40 subjects) including skin reddening, skin lesions, skin abrasions, a blood blister, chafing, skin irritation due to electromyography (EMG) electrodes, and bruises (Husemann et al., 2007; Borggraefe et al., 2010; Vaney et al., 2012; Kelley et al., 2013a,b; Nilsson et al., 2014; Stoller et al., 2014; Esquenazi et al., 2017; Jansen et al., 2018). The most severe AE reported was one bone fracture in the context of Lokomat training (Filippo et al., 2015). The fracture to the proximal anterior and medial part of the tibia occurred in a patient with T12 incomplete paraplegia. The authors did not report any unusual event causing the injury. The patient had trained 18 sessions in the Lokomat (30 min per session, 5 times per week, 50% BWS, guiding force between 75 and 100%) and complained of pain in the anterior region of the knee at the beginning of session 19 (Filippo et al., 2015). Bone densitometry performed after the event revealed low bone mineral density. The result of this did not classify as severe osteoporosis, which would have constituted a contraindication for Lokomat training. The two other severe AEs were an open skin lesion and a tendinopathy during Lokomat training (Borggraefe et al., 2010). Mild or moderate joint pain was reported 21 times and occurred mostly in the knee (Freivogel et al., 2008, 2009; Borggraefe et al., 2010; Morone et al., 2011; Geigle et al., 2013; Nilsson et al., 2014). Other musculoskeletal AEs included muscle pain, tendinopathy, and low back pain (Freivogel et al., 2008; Borggraefe et al., 2010), totaling 40 occurrences. There were 21 physiological AEs in 7 subjects including giddiness (mild) (Chin et al., 2010), and blood pressure changes (both hypotension and hypertension) (Morone et al., 2011; Geigle et al., 2013; Stoller et al., 2014; Ikumi et al., 2016) that were all classified as moderate (Figure 2). Another frequent AE (more than 17 occurrences) was discomfort related to the harness (e.g., to the groin, armpit, or shoulders) that was mostly classified as mild (Freivogel et al., 2008, 2009; Chin et al., 2010; Carda et al., 2012; Geigle et al., 2013; Nilsson et al., 2014; Stoller et al., 2014). Other AEs identified in this review with seven occurrences were pain (not specified) (Esquenazi et al., 2017), fear of the gait robot (Chin et al., 2010), headache, menstrual cramps (Freivogel et al., 2008), the feeling of being trapped, and the sense of the gait robot being heavy over the lower back (Nilsson et al., 2014).

**Table 3:** Adverse events detailed.

**References**	**Device**	**Adverse event description**	**AE occurrence**	**Severity**	**Category AE**	**Caused by**	**Caused dropouts**
Borggraefe et al. (2010)	Lokomat	Muscle pain	16	Mild	Musculoskeletal	Not stated	No
		Joint pain	14	Mild (12), moderate (2)	Musculoskeletal	Not stated	Yes (2)
		Skin erythema	12	Mild	Soft tissue-related	Cuffs	No
		Open skin lesions	4	Mild (2), moderate (1), severe (1)	Soft tissue-related	Cuffs	Yes (2)
		Tendinopathy	1	Severe	Musculoskeletal	Not stated	Yes
Carda et al. (2012)	Lokomat	Mild discomfort	3	Mild	Soft tissue-related	Harness	No
Chin et al. (2010)	Lokomat	Discomfort and redness in groin area	A few	Mild	Soft tissue-related	Harness	No
		Skin abrasions	3	Moderate	Soft tissue-related	Cuffs	No
		Giddiness	1	Mild	Physiological	Not stated	No
		Lower limb bruises	1	Moderate	Soft tissue-related	Cuffs	Yes
		Fear of gait trainer	1	Moderate	Other	Not stated	Yes
Esquenazi et al. (2017)	Not stated (Lokomat or G-EO)	Skin irritation and pain	4	Unknown	Soft tissue-related/other	Not stated	No
Filippo et al. (2015)	Lokomat	Proximal tibia fracture	1	Severe	Musculoskeletal	Not stated	Not applicable
Freivogel et al. (2008)	LokoHelp	Discomfort in groin or armpit	11	Mild	Soft tissue-related	Harness	No
		Discomfort in right hip	1	Mild	Musculoskeletal	Not stated	No
		Lower back pain	1	Mild	Musculoskeletal	Not stated	No
		Headache	3 (1)^a^	Mild	Other	Not stated	No
		Menstrual cramps	1	Mild	Other	Not stated	No
		Knee pain	1	Moderate	Musculoskeletal	Not stated	No
		Not described	5	Unknown	Unknown	Not stated	No
Freivogel et al. (2009)	LokoHelp	Discomfort	33	Unknown	Soft tissue-related	Mostly harness	No
		Knee pain	1	Unknown	Musculoskeletal	Not stated	No
Geigle et al. (2013)	Lokomat	Atypical autonomic dysreflexia	4 (1)^a^	Moderate	Physiological	Exercise (did not occur during pure suspension)	Yes (dropped out due to elevated BP)
		Knee pain	1	Minor	Musculoskeletal	Not stated	
		Discomfort	1	Minor	Soft tissue-related	Harness	
Husemann et al. (2007)	Lokomat	Skin lesions	2	Moderate	Soft tissue-related	Not stated	Yes
Ikumi et al. (2016)	HAL	Transient blood pressure change	6 (1)^a^	Moderate	Physiological	Not stated	No
Jansen et al. (2018)	HAL	Skin reddening	4	Mild	Soft tissue-related	EMG electrodes, leg cuffs, shoes	No
Kelley et al. (2013a,b)	Lokomat	Skin changes (redness or broken skin)	12 (5)^a^	Moderate	Soft tissue-related	Straps/cuffs	No
Morone et al. (2011)	GT	Severe symptomatic hypotension	8 (3)^a^	Moderate	Physiological	Not stated	Not stated
		Knee pain	1	Moderate	Musculoskeletal	Not stated	Not stated
Nilsson et al. (2014)	HAL	Knee/malleolus pain	2	Moderate	Musculoskeletal	Cuff pressure	No
		Discomfort (feeling of being trapped)	1	Moderate	Other	Straps	No
		Discomfort (shoulders)	2	Mild	Soft tissue-related	Straps	No
		Sense of suit being heavy over lower back	1	Mild	Other	Weight of suit	No
		Skin irritation	1	Mild	Soft tissue-related	EMG electrodes	No
		Groin pain, chafing	1	Moderate	Soft tissue-related	Harness	No
Stoller et al. (2014, 2015)	Lokomat	Tibia skin lesion	1	Moderate	Soft tissue-related	Cuffs (padding)	Yes
		Groin pain	1	Moderate	Soft tissue-related	Harness	Yes
		High blood pressure	2 (1)^a^	Moderate	Physiological	Not stated	No
Vaney et al. (2012)	Lokomat	Minor bruising	Some	Mild	Soft tissue-related	Straps	No

a *When AE occurrence is presented as X (Y), X is the number of events, and Y is the number of subjects*.

There was limited information available on the duration of gait training before an AE occurred. Chin et al. (2010) stated that the dropouts due to bruises and fear of the Lokomat system occurred after 2–5 training sessions of 15–45 min each, and the tibia fracture (Filippo et al., 2015) occurred after 18 sessions of 30 min each. Knee pain in LokoHelp (Freivogel et al., 2008) occurred after 4 sessions of 30 min. Autonomic dysreflexia during Lokomat training (Geigle et al., 2013) occurred 20 min into the 10th training session after having completed 9 40-min sessions, and transient blood pressure change in HAL training (Ikumi et al., 2016) was observed 6 times in 10 sessions of 60 min including preparation time. In a case report on the management of skin injuries during Lokomat training (Kelley et al., 2013b), it is reported that the subject walked a total of 2 h in 5 sessions in the Lokomat before the first injury was observed. Borggraefe et al. (2010) found no correlation between AE incidence and age, duration of RAGT, number of sessions, or total distance walked. They did, however, report that both obese children included in the study developed soft tissue-related AEs (skin erythema, open skin lesion) and that in two cases, skin lesions developed next to skin areas covered by diapers.

Methods used to detect AEs included documentation of patient feedback or complaints (Freivogel et al., 2008, 2009; Borggraefe et al., 2010; Geigle et al., 2013; Nilsson et al., 2014; Stoller et al., 2014, 2015; Filippo et al., 2015), patient questionnaires (Borggraefe et al., 2010), MRI for the detection of a fracture (Filippo et al., 2015), blood pressure monitoring (Geigle et al., 2013; Stoller et al., 2014, 2015; Ikumi et al., 2016), and medical screening before, after, and when needed during each training session (Kelley et al., 2013a,b).

Table 4 summarizes the frequencies of injury types, severities, and causes in the different devices. More than 169 AEs were reported in more than 79 subjects. Exact numbers cannot be stated as the description of AEs was incomplete in four studies (Freivogel et al., 2009; Chin et al., 2010; Vaney et al., 2012; Esquenazi et al., 2017). Therefore, the occurrences are displayed as ranges in this table. In graphical representations and further analysis of this data, the minimum numbers will be used and presented. In total, between 8 and 13% of the participants experienced AEs. For the Lokomat users, this was between 12 and 18%, for the LokoHelp users between 18 and 90%, for the HAL users 9%, for the GT users 1%, and for the G-EO users between 0 and 57%.

**Table 4:** Adverse events classified per device.

	**Total**	**Lokomat**	**LokoHelp**	**HAL**	**GT**	**G-EO**
**Event types**						
Soft tissue-related	>96^a^^,^^b^^,^^c^^,^^d^	>40^a^^,^^b^^,^^d^	44^c^	8	–	≤4^b^
Musculoskeletal	40	33	4	2	1	–
Physiological	21	7	–	6	8	–
Other	7	1	4	2	–	–
Not specified	5	–	5	–	–	–
**Event severity**						
Mild	>73^a^^,^^d^	>48^a^^,^^b^^,^^d^	17	8	–	–
Moderate	50	30	1	10	9	–
Severe	3	3	–	–	–	–
Unknown	43^c^	≤4^b^	39^c^	–	–	≤4^b^
**Part of device causing AE**						
Cuffs/straps	>42^d^	>33^d^	–	9	–	–
Harness	>50^a^	>5^a^	≤44^c^	1	–	–
Total no. of events	>169^a^^,^^d^	>81^a^^,^^b^^,^^d^	57	18	9	≤4^b^
Total no. of subjects	79<*n*≤124^a^^,^^b^^,^^d^	57<*n*≤90^a^^,^^b^^,^^d^	4<*n*≤20^d^	10	4	≤4^b^

a *“A few patients experienced discomfort and developed redness in their groin area”; unknown how many patients/events (Chin et al., 2010)*.

b *“4 reported adverse events that were study related due to skin irritation and pain”; unknown whether adverse events occurred in Lokomat or G-EO training, and how many subjects were affected (Esquenazi et al., 2017)*.

c *34 complaints, unclear by how many of the 16 subjects; “mostly” related to the harness (soft tissue) (Freivogel et al., 2009)*.

d *“Some minor bruising from the straps”; number of affected subjects/events not stated (Vaney et al., 2012)*.

The chi-squared tests indicated that there is no independence of variables in all tested combinations: devices and reported AE types (χ^2^ = 88.05, *p* < 0.01), device types and reported AE types (χ^2^ = 15.88, *p* < 0.01), AE types and severity level (χ^2^ = 75.70, *p* < 0.01), devices and severity level (χ^2^ = 115.05, *p* < 0.01), and device types and severity level (χ^2^ = 70.80, *p* < 0.01). We can therefore conclude that there are relationships between devices, device types, AE types, and severity levels of AEs. In other words, the occurrence of AE types differs between device types and between devices, as does the severity between devices, device types, and AE types. Articles that did not state absolute numbers (Chin et al., 2010; Vaney et al., 2012; Esquenazi et al., 2017) were excluded from this analysis.

**Figure 3:**
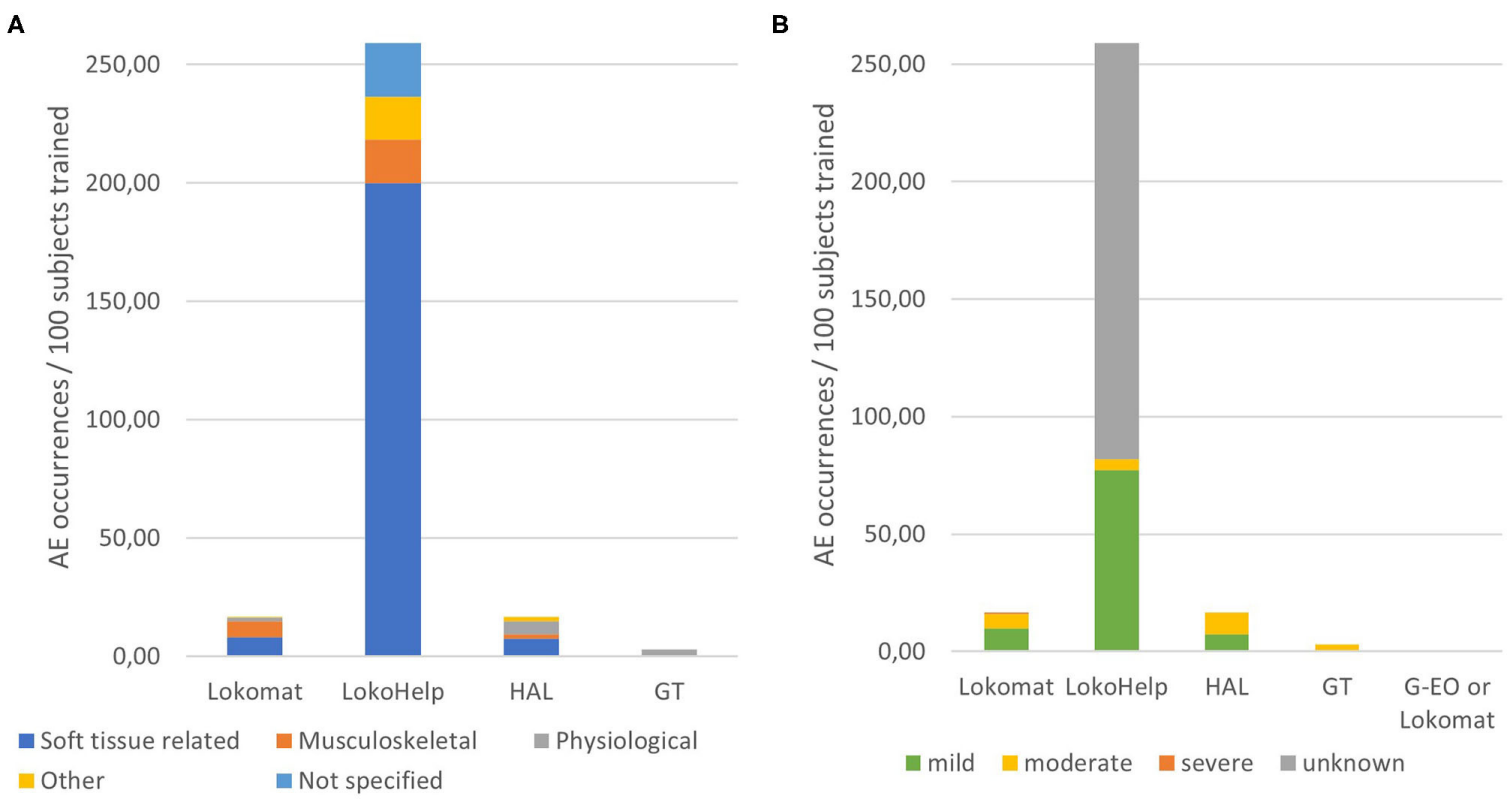
Distributions of adverse event types and severities per devices. **(A)** Occurrences of adverse event types relative to the total number of subjects trained in each device. **(B)** Occurrences of adverse event severities relative to the total number of subjects trained in each device.

Relations of AE severity and AE types with device types and devices are detailed in Figure 3. Relative to the total number of subjects that trained in each of the devices, on average, 16.6 AE occurrences per 100 subjects were reported for Lokomat training, 259 occurrences per 100 subjects for LokoHelp training, 16.7 occurrences per 100 subjects for HAL training, and 3 occurrences in 100 subjects for GT training. While there were no physiological AEs reported in LokoHelp and only physiological AEs in GT, subjects training in the two exoskeleton-type devices Lokomat and HAL were reported to have experienced soft tissue-related, musculoskeletal, and physiological AEs (Figure 3A). The overall severity of AEs in GT was the highest (4.00) with all AEs being moderate, followed by HAL (2.67), Lokomat (2.44), and LokoHelp (1.17) with the majority of AEs being mild (Figure 3B). Regarding AE types, physiological AEs had the highest overall severity (3.86), followed by soft tissue-related AEs (2.27) and other AEs (1.86). Musculoskeletal AEs had the lowest overall severity of 1.92.

## Discussion

In this systematic literature review, we extracted and analyzed information on AEs in RAGT from 50 included studies, involving 985 subjects in total. AEs occurred in 36% of the included studies and in 8–13% of the subjects. The findings show that skin injuries and a bone fracture occurred in RAGT, supporting our hypothesis. Moreover, a substantial amount of reports of joint pain, blood pressure change, and discomfort caused by the harness indicates that injuries associated with RAGT are broader than skin damage and bone fractures.

The most frequently reported AEs (>96 occurrences, constituting more than half of all AEs) were injuries or discomfort to the skin or underlying tissue (Husemann et al., 2007; Borggraefe et al., 2010; Vaney et al., 2012; Kelley et al., 2013a,b; Nilsson et al., 2014; Stoller et al., 2014, 2015; Esquenazi et al., 2017; Jansen et al., 2018), joint pain (21 occurrences) (Freivogel et al., 2008, 2009; Borggraefe et al., 2010; Morone et al., 2011; Geigle et al., 2013; Nilsson et al., 2014), blood pressure change (20 occurrences) (Morone et al., 2011; Geigle et al., 2013; Stoller et al., 2014, 2015; Ikumi et al., 2016), and discomfort related to the harness (more than 20 occurrences) (Freivogel et al., 2008, 2009; Chin et al., 2010; Carda et al., 2012; Geigle et al., 2013; Nilsson et al., 2014; Stoller et al., 2014, 2015). Next to a tendinopathy and an open skin lesion (Borggraefe et al., 2010) classified as severe AEs, the most severe AE (and only SAE) was a tibia fracture (Filippo et al., 2015).

### Occurrence and Severity of AEs

The overall severity of physiological AEs was the highest (3.86), which is related to the fact that training is usually interrupted when a sudden blood pressure change occurs. While one might expect that musculoskeletal AEs are generally more severe than soft tissue-related AEs, the overall severity of musculoskeletal AEs (1.92) was slightly lower than that of soft tissue-related AEs (2.27). Mild musculoskeletal AEs were minor pain or discomfort to the joints or muscles. There were 22 moderate and 1 severe soft tissue-related AEs that included open skin lesions (one of which was severe), bruises, and groin pain. For 37 soft tissue-related events, no severity could be inferred from the reported information that might have an influence on the overall severity. It can, however, be concluded that not only physiological and musculoskeletal but also soft tissue-related AEs can require interrupting the RAGT or even medical attention. Specifically in subjects with restricted blood flow or reduced sensation, complications can arise from smaller skin or soft tissue injuries, and healing can be impaired (Bader et al., 2019), which can explain the relatively high overall severity of soft tissue-related AEs. Remarkably, in studies that included healthy subjects, no AEs were experienced, which supports the notion that disturbed physiological and/or sensory function in patients could be a relevant factor. This implies that risks for soft tissue-related AEs should be taken just as seriously as risks for musculoskeletal AEs.

Regarding the devices, the largest absolute number of AEs was reported for training in Lokomat (more than 81 events in 57–90 subjects). However, one has to keep in mind that the 50 included articles included 27 Lokomat studies with 489 subjects. So, per 100 subjects, an average of 16.6 AEs was reported in Lokomat training. This is comparable with 18 AEs in 108 subjects performing RAGT with HAL resulting in an average of 16.7 AEs per 100 subjects. For GT, an average of 3.0 AEs per 100 subjects was reported (9 AEs in 301 subjects) and for G-EO between 0 and 57.1 events per 100 subjects. By far, the highest relative AE occurrence was reported for LokoHelp training, where the reports (57 AEs in 22 subjects) result in an average of 259 AEs per 100 subjects. Interestingly, while LokoHelp training resulted in the highest relative number of AEs, it also resulted in the lowest overall severity (1.17). Lokomat and HAL are comparable not only in occurrence but also in overall severity of AEs (2.44 and 2.67, respectively). All AEs reported in relation with GT training were moderate (overall severity 4).

### Risk Factors

The results of the analyses suggest that AEs do occur in RAGT, independent of the subjects' age and diagnosis. There were no striking differences in the level of severity or time since onset of the disease. We did, however, observe that there were no reports of AEs in healthy participants. This could be due to a number of reasons. Firstly, only 14 out of 985 subjects (1.4%) were healthy individuals. Secondly, RAGT with healthy individuals was only performed during 1 day in each of the studies (Gizzi et al., 2012; Asbeck et al., 2015; Wu et al., 2015), as they are not the targeted population for a training program to improve walking. Both of these aspects decrease the chance of suffering an injury. Thirdly, the characteristics of certain patient groups, such as restricted blood flow, reduced sensation, uncontrolled muscle activities, or reduced bone mineral density, might increase the risk of sustaining injuries in RAGT, in contrast to healthy individuals. The current findings allow us to identify which risk factors are most likely involved in the various AEs reported during stationary RAGT in patients.

#### Soft Tissue-Related Adverse Events

According to the results, skin, and other soft tissue injuries are the most frequent AEs related to RAGT. They are mostly caused by either the cuffs/straps (Borggraefe et al., 2010; Chin et al., 2010; Vaney et al., 2012; Kelley et al., 2013a,b; Nilsson et al., 2014; Stoller et al., 2014, 2015; Jansen et al., 2018) or the harness (Freivogel et al., 2008, 2009; Chin et al., 2010; Carda et al., 2012; Geigle et al., 2013; Nilsson et al., 2014; Stoller et al., 2014, 2015) and occurred in both device types, although slightly more frequently in end-effector-type devices (13.5 occurrences per 100 subjects) than in exoskeleton-type devices (7.7 occurrences per 100 subjects on average). Both the cuffs/straps and the harness are mentioned as causes for soft tissue injuries in seven unique studies, respectively. In addition to that, one article mentions diapers as well as obesity as possible risk factors for skin injuries (Borggraefe et al., 2010). Remarkably, issues related specifically to cuffs or straps have only been reported in exoskeleton-type devices (Lokomat and HAL). End-effector-type devices are only attached to the foot and sometimes to the shank, which decreases the number of contact interfaces between human (skin) and robot, reducing the chances for skin irritation at the cuffs and straps in end-effector-type devices. In contrast, exoskeletons have a risk of misalignment between joint axes, which can lead to displacements of the cuff relative to the human limb, resulting in increased shear and pressure in the interface between cuff or strap and skin, which can contribute to soft tissue injuries (Rocon et al., 2008; Akiyama et al., 2015; Mao et al., 2015).

The harness has been stated to be the cause of AEs in Lokomat (>5 AEs) (Chin et al., 2010; Carda et al., 2012), HAL (1 AE) (Nilsson et al., 2014), and LokoHelp (44 AEs) (Freivogel et al., 2008, 2009) with 88% of the events related to the end-effector-type device LokoHelp. The affected body regions included the groin area (Freivogel et al., 2008; Chin et al., 2010; Nilsson et al., 2014; Stoller et al., 2014) and armpits (Freivogel et al., 2008). One might assume that higher percentages of BWS lead to a higher risk of discomfort or injuries related to the harness because the pressure in the interface harness–skin is increased. The range of documented BWS in end-effector-type devices was between 0 and 50% and in exoskeleton-type devices between 0 and 100%. It is striking that all studies with BWS above 50% of body weight reported AEs. In the studies reporting discomfort due to the harness, the maximum BWS ranged between 30 and 100% of body weight. All studies reporting BWS above 55% also reported discomfort related to the harness, with the exception of one case report (Kelley et al., 2013b) where only the first session was started at 100% BWS but as of the end of session 1, BWS was always <50%. Nevertheless, the large number of harness-related AEs in LokoHelp training was reported in two studies with BWS under 30% (Freivogel et al., 2008, 2009). Therefore, lower BWS might decrease but not completely avoid the risk of discomfort related to the harness. Other possible factors might be the design, fitting, or material of the harness as well as the clothes worn by the subjects (Rocon et al., 2008; Kelley et al., 2013b).

Overall, the susceptibility to soft tissue-related AEs could be influenced by harness or cuff design and fit, subject characteristics, and materials involved in the cuff–skin interface. One study analyzed this aspect and reported that there was no correlation between the incidence of AEs and age (Borggraefe et al., 2010), but that both obese children included in the study developed a soft tissue-related AEs. Moreover, they observed two open skin lesions adjacent to the area where diapers were worn. Another study reported that wrapping the legs of a subject presenting with thin and flaky skin with viscoelastic polymer sheets and elastic bandages helped manage soft tissue-related AEs (Kelley et al., 2013b). Therefore, in addition to the fit of cuffs and harness, both the subjects' weight and/or body composition and materials present in the interface between skin and robot cuffs or the harness might alter the risk for soft tissue-related AEs.

#### Musculoskeletal Adverse Events

The findings of this review show that RAGT can lead to musculoskeletal injuries, such as a bone fracture and joint pain. Musculoskeletal AEs were reported in relation to training in Lokomat, LokoHelp, GT, and HAL and therefore in both exoskeleton-type devices and end-effector-type devices. However, 88% of the reported musculoskeletal AEs occurred in an exoskeleton-type device (on average 5.6 musculoskeletal AEs per 100 subjects in exoskeleton-type devices compared with 1.4 musculoskeletal AEs per 100 subjects in end-effector-type devices). This leads to the assumption that the risk of sustaining a musculoskeletal injury is higher during exoskeleton-type RAGT. However, it is possible that this is influenced by a single study reporting many occurrences of musculoskeletal AEs (31) in one exoskeleton-type device (Borggraefe et al., 2010). The only SAE (bone fracture) reported in the included articles occurred in an exoskeleton-type device. To the best of our knowledge, there are no reports of bone fractures in end-effector-type devices. While this was the only occurrence of a bone fracture in RAGT found through this review, there are several reports of bone fractures in overground exoskeletons (He et al., 2017; van Herpen et al., 2019). Misalignment is frequently mentioned as the assumed cause for bone fractures in overground exoskeleton devices (He et al., 2017; van Herpen et al., 2019), but this has not been discussed as a possible cause in the case report of the tibia fracture sustained during Lokomat training (Filippo et al., 2015). The authors of this case report discussed low bone mineral density as a possible influencing factor but did not report any details of the relevant training session or discussed other possible reasons. Due to the oversimplification of exoskeleton joints compared with anatomical joints, misalignments are unavoidable (Rocon et al., 2008; Akiyama et al., 2012). This might lead to the assumption that end-effector-type devices are inherently safer than exoskeleton-type devices. However, end-effector-type devices provide less guidance of the movements and could therefore create movements in arbitrary directions and excessive moments that can cause considerable harm (Rocon et al., 2008). In end-effector-type gait trainers, this risk could be mitigated by providing appropriate BWS. However, in all end-effector-type gait trainer studies included in this review, BWS was reported to be 50% or lower, whereas exoskeleton-type studies reported BWS up to 100%. This is not related to less severely affected subjects being involved in end-effector-type RAGT studies. The subjects in studies with both types of gait trainers varied strongly in disease severity and walking ability.

#### Physiological Adverse Events

Giddiness and changes in blood pressure were reported in relation to Lokomat, HAL, and GT training (Chin et al., 2010; Morone et al., 2011; Geigle et al., 2013; Stoller et al., 2014, 2015; Ikumi et al., 2016). There were 13 occurrences in 4 subjects reported in exoskeleton-type devices and 8 occurrences in 3 subjects reported in end-effector-type devices. It is striking that all reported blood pressure changes occurred in more severely affected subjects with SCI [American Spinal Injury Association (ASIA) A and C] or subacute stroke [functional ambulation category (FAC) <3]. This indicated that the risk for blood pressure changes in RAGT might be increased in subjects who do not ambulate independently. It is also worth noting that hypertension in SCI as a result of autonomic dysreflexia seems to be linked to the stepping movements in combination with the upright position and did not occur during pure suspension in the harness (Geigle et al., 2013). A close blood pressure monitoring of patients with a history of blood pressure changes or high risks of orthostatic hypotension or autonomic dysreflexia could help mitigate the risk of physiological AEs.

### Documentation of Adverse Events

The documentation of AEs lacks detail in most studies. A significant amount of included articles (36%) did not provide a complete description of AEs, even though the requirements for regarding AE documentation as complete were relatively low: description of events, the number of affected subjects, and the associated device. The assumed cause of the event was only stated in about half of the reports. Moreover, there is a need for documentation on how different types of AEs can be managed or avoided (Kelley et al., 2013b).

Although we did not consider this as critical for documenting AEs, it is striking that most reports did not include any information on the duration of training before the AEs occurred or the characteristics of the affected subjects. Training time before the occurrence of an AE was often not stated. Based on the literature, one could assume that skin-related AEs are more likely to occur in the first training sessions as the skin can habituate to the stress (Sanders et al., 1995; Yandell et al., 2020). In the two studies stating the durations of training before the soft tissue-related AEs, they occurred between session 2 and 5 (Chin et al., 2010; Kelley et al., 2013b). A more detailed analysis of this aspect is not possible as there is not enough information available on whether the complaints occurred in the beginning or end of the sessions and due to the fact that most studies did not report on training time before AE onset. Subject characteristics related to AEs were only analyzed in detail in one of the included studies (Borggraefe et al., 2010). In order to establish more generalizable relations between subject or training characteristics and risk factors, more detailed reports of those aspects in relation to AEs are needed.

Structural documentation of AEs related to RAGT (or any medical device for that matter) is currently not optimally supported or facilitated by regulatory bodies. In other words, AE reporting is not sufficiently obligatory and public. Although some information on safety is shared through the reporting system of the FDA in the US, reporting is only mandatory if it is an SAE and only for manufacturers and healthcare institutions, but not for individual healthcare professionals and consumers (Maak and Wylie, 2016). In the EU, there is currently no central reporting system. There are obligations for the manufacturers to report AEs to the competent authorities on a national level, but this information is currently not shared with the public. In relation to the current transition from the EU Medical Device Directive to Medical Device Regulation, the reporting system EUDAMED is expected to be (re-)launched in May 2022, with more firm rules for reporting. Information on SAEs, device deficiencies, vigilance, and post-market surveillance is intended to be submitted through this platform, which will be partly open to the public. Dissemination will include information on device safety and issued certificates, vigilance, and post-market surveillance (European Commission, 2019), although the exact extent to which information will be accessible to whom is currently unknown. Based on the outcomes of the current review, such facilities are needed to allow and stimulate a more structural reporting of and access to AEs (and not only SAEs). This cannot only inform the end user of risks associated with a certain device but also encourage new, safety-related developments, and ultimately improve safety of RAGT.

### Limitations

The findings of this systematic literature review need to be interpreted with care for several reasons. The primary outcome of the study is AEs reported in RAGT, which is why terms related to AEs and injuries were included in the search query. However, most bibliographic databases search for the entered search terms in titles, keywords, and abstracts of articles. During this process, we found that information on AEs is frequently not contained in those elements but in the body of the text, complicating the search for relevant articles. Therefore, we also searched full-text databases, but we cannot be sure that we identified all relevant articles with that method.

Another limitation of this review is a potential overlap of studies. We excluded double reports as much as possible, but we cannot rule out that some articles contained information on the same participants in the same experiment without stating this (e.g., data from a case report on a specific AE might be part of a clinical trial too). Furthermore, some of the relationships between AE occurrence and device type could be biased by few studies stating many AEs for one specific device (Freivogel et al., 2008, 2009; Borggraefe et al., 2010) or including vague statements, such as only reporting on (the absence of) SAE or not specifying AE occurrence for each of the interventions involved (Chin et al., 2010; Wu et al., 2015; Esquenazi et al., 2017).

Other limitations are related to an expected underrepresentation and incomplete documentation of AEs. It is possible that many other studies where no AEs occurred were published but not included in this article, if they did not contain a statement about AE occurrence. Moreover, we noticed strong variations in the level of detail in which AEs are recorded during a study and reported in articles: while some articles only include more obvious or severe AEs, others may mention all cases of slight discomfort and have asked participants specifically about their experience. The high relative occurrence of AEs in LokoHelp, but with the lowest overall severity, is a likely example of this. This hampered a reliable comparison of AE occurrence and severities between device types or devices. More detailed descriptions of AEs and their effects with regard to the interruption of training or the needed medical attention would allow for a more accurate and detailed severity rating, thereby enabling more valid comparisons. We therefore suggest that editors focus on a correct and complete statement on AEs in scientific reports on medical devices. A statement saying that there were no AEs is just as important as a detailed description on occurred AEs to learn about the risks associated with a device. The extension to the CONSORT statement (Schulz et al., 2010) for reporting of harms in randomized controlled trials (Ioannidis et al., 2004) could serve as a guideline for this. While the CONSORT statement is specifically designed for improving reporting in randomized controlled trials, we suggest that the checklist for reporting of harms is also relevant for other study types. Based on the experiences collected in the process of this systematic literature review, we would like to encourage a focus on the following aspects when reporting on AEs in medical device trials:

- Collection of AE information: how were numbers of AEs obtained? Who reported them and were any questionnaires or procedures involved?
- Documentation of AE information: are all AEs reported or only a specific subset? Report both number of affected subjects and number of occurrences per subject. If no AE occurred, this should be stated clearly.
- AE descriptions: describe the observed AE concisely including the location. Describe unusual events or subject characteristics that might be related to the AE and discuss possible reasons.
- AE consequences: did the intervention have to be interrupted? For how long? Was medical attention required? Did the AE cause a dropout and who made that decision? Preferably use standardized definitions of severity levels.

### Implications for the Use of Rehabilitation Robots

The aim of this review is to raise awareness of the safety of rehabilitation robots, and while it focuses on the risks and needs of rehabilitation robots, it is not intended to discourage their use. Although AEs do occur in RAGT, it has positive effects on gait and has potential to decrease the burden on healthcare professionals (Freivogel et al., 2009; Hesse and Werner, 2009; Mehrholz et al., 2017). Therefore, a proper balancing of risks and benefits is needed, but in order to do this, proper information about AEs is needed as part of ethical and regulatory decisions to allow the use of rehabilitation robots in clinical practice. In order to do this well, correct and sufficient information about AEs is needed. Moreover, AEs should not only be documented but also be disseminated to raise awareness of risks. The need for information flow goes both ways: manufacturers should make their risk/benefit weighting more transparent to allow for healthcare professionals ideally to make an informed decision on the use of robotic devices in therapy, in-/exclusion criteria, associated risks, and possible measures. In return, healthcare professionals and researchers should report on AEs and their management, where applicable, in a structured and systematic way to inform developers of rehabilitation robots about ways to improve safety of their devices.

### Conclusions

In the present systematic literature review on AEs during the use of stationary robotic gait trainers, including 50 studies and 985 subjects, we found that a total of 169 AEs occurred in 36% of the studies, affecting between 8 and 13% of the subjects. The most frequent types of AEs were soft tissue-related AEs and musculoskeletal AEs, whereas physiological AEs had the highest overall severity, followed by soft tissue-related AEs. Soft tissue-related AEs occurred slightly more frequently in end-effector-type devices than in exoskeleton-type devices and were often associated with the cuffs or straps (only mentioned in relation to exoskeleton-type devices) or with the harness (mostly mentioned in relation to end-effector-type devices). Musculoskeletal AEs were reported more frequently in exoskeleton-type devices than in end-effector-type devices. We have identified two main risk factors: forces in the skin–robot interface causing skin injuries and forces on the musculoskeletal level causing pain or injuries to the musculoskeletal system. On a more detailed level, hazards are most likely related to an incorrect model fit, insufficient compliance at the points of force transmission from robot to human, materials present at the human–robot interface, misalignments of rotation axes, or subject characteristics, such as uncontrolled muscle activities or susceptibility to injuries due to overall health status. We additionally identified a lack of completeness of AE reporting in RAGT studies and would like to stress the need for accurate and complete documentation and dissemination of AEs for the identification of hazards and possible mitigation measures. Therefore, AE documentation should receive more attention, and researchers, relevant authorities, as well as journal editors should ensure the appropriate documentation and dissemination of RAGT-related AEs.

The present findings suggest that future developments in RAGT should focus on the subjects' safety, especially mitigating risks associated with pressure and shear applied to the subject's skin, as well as forces applied to the musculoskeletal system that can be harmful due to misalignments. To further investigate the effects of these hazards, appropriate measurement methods and experiments are needed. Further, the investigation of forces present in the human–robot interface as well as investigations on acceptable limit values for comfort and safety could help to establish best practices for safe use of rehabilitation robots.

## Data Availability Statement

The original contributions presented in the study are included in the article/supplementary material, further inquiries can be directed to the corresponding author/s.

## Author Contributions

JBu, GP-L, LS, and JBe contributed to conception and design of the study. EP, GP-L, RS, and JBe contributed to the literature screening process. JBe performed the data analysis and wrote the first draft of the manuscript. GP-L wrote sections of the manuscript. All authors contributed to manuscript revision, read, and approved the submitted version.

## Conflict of Interest

The authors declare that the research was conducted in the absence of any commercial or financial relationships that could be construed as a potential conflict of interest.

## Appendix

### Search Strategy PubMed

#1 robotics [MeSH] OR robot-assisted OR robotics-assisted OR electromechanical OR electro-mechanical
#2 exercise therapy [MeSH] OR rehabilitation OR training
#3 gait OR walk OR walking OR step OR stepping OR locomotor OR locomotion
#4 #1 AND #2 AND #3
#5 “body weight support” OR “body weight supported”
#6 #5 AND “treadmill training”
#7 #4 OR #6 OR “locomotor training” OR Lokomat OR Gangtrainer OR G-EO OR WALKBOT OR LokoHelp
#8 adverse OR “skin breakdown” OR “skin lesion” OR “skin sore” OR “pressure sore” or discomfort OR abrasion
#9 #7 AND #8
